# 

**Published:** 1883-07

**Authors:** 


### Pamphlets.

---

Alcohol as a Food, a Medicine, and as a Luxury. By Geo. C. Pitzer, M D., Prof. of the theory and practice of medicine in the American Medical College, St. Louis, Mo., etc., etc.

The Proceedings of the Medical Society of the county of Kings, New York.

A case of Hysteredectomy with a New Clamp, for the Removal of Large Uterine tumors. By H. P. C. Wilson, M.D., of Baltimore. Gynecologist for St. Vincent's Hospital and the Union Protestant Infirmary, etc., etc.

The Official Correspondence Between Surgeon-General Wm. A. Hammond, U. S. A. and the Adjutant-General of the Army, Relative to the Founding of the Army Medical Museum, and the Inauguration of the Medical and Surgical History of the War. D. Appleton & Co., N. Y.

A Year's Work in Ovariotomy. By William Goodell, M.D., Prof. clinical gynecology in the University of Pennsylvania. From the *Med. News*, April 14, 1880.

Surgical Treatment of Wounds. By R. H. Day, M.D. Read before the Louisiana State Med. Society, in Shreveport, April, 1883.

The Pathology and Morbid Anatomy of tubercle. Report to the Wisconsin State Medical Society. By N. Senn, M.D., of Milwaukee.

Cancer of the Intestinal Tract: Operations for the Removal of Malignant Strictures of Pylorus and Intestines, Together with a Brief Review of the Historical Development of Modern Abdominal Surgery. By Reuben Vance, M.D., Prof. of operative surgery and clinical surgery in the medical department of the University of Wooster, Cleveland, Ohio.

The Symptoms of Diagnosis of Malaria in Children. By L. Emmett Holt, A.M., M.D., New York. Attending Physician to the Northwestern Dispensary in the Department of Diseases of Children. Reprinted from the *American Journal of obstetrics.*

